# Computational fluid dynamics comparison of prevalent liquid absorbents for the separation of SO_2_ acidic pollutant inside a membrane contactor

**DOI:** 10.1038/s41598-023-28580-6

**Published:** 2023-01-24

**Authors:** Yan Cao, Ali Taghvaie Nakhjiri, Mahdi Ghadiri

**Affiliations:** 1 School of Computer Science and Engineering, Xi’an Technological University, Xi’an, 710021 People’s Republic of China; 2 Department of Petroleum and Chemical Engineering, Science and Research Branch, Islamic Azad University, Tehran, Iran; 3 Institute of Research and Development, Duy Tan University, Da Nang, 550000 Vietnam; 4 The Faculty of Environment and Chemical Engineering, Duy Tan University, Da Nang, 550000 Vietnam

**Keywords:** Chemical engineering, Environmental impact, Environmental chemistry

## Abstract

In recent years, the emission of detrimental acidic pollutants to the atmosphere has raised the concerns of scientists. Sulphur dioxide (SO_2_) is a harmful greenhouse gas, which its abnormal release to the atmosphere may cause far-ranging environmental and health effects like acid rain and respiratory problems. Therefore, finding promising techniques to alleviate the emission of this greenhouse gas may be of great urgency towards environmental protection. This paper aims to evaluate the potential of three novel absorbents (seawater (H_2_O), dimethyl aniline (DMA) and sodium hydroxide (NaOH) to separate SO_2_ acidic pollutant from SO_2_/air gaseous stream inside the hollow fiber membrane contactor (HFMC). To reach this goal, a CFD-based simulation was developed to predict the results. Also, a mathematical model was applied to theoretically evaluate the transport equations in different compartments of contactor. Comparison of the results has implied seawater is the most efficient liquid absorbent for separating SO_2_. After seawater, NaOH and DMA are placed at the second and third rank (99.36% separation using seawater > 62% separation using NaOH > 55% separation using DMA). Additionally, the influence of operational parameters (i.e., gas and liquid flow rates) and also membrane/module parameters (i.e., length of membrane module, hollow fibers’ number and porosity) on the SO_2_ separation percentage is investigated as another highlight of this paper.

## Introduction

In recent years, combustion of fossil fuels is known as one of the momentous reasons of human-based greenhouse gases emission to the atmosphere^1–3^. Sulphur dioxide (SO_2_) is an important greenhouse gas, which its release into the atmosphere may result in various adverse effects on human health and ecosystem including respiratory problems, asthma attacks, urban smog and acid rain^4,5^. By the emission of this acidic gas to the atmosphere, sulfur trioxide (SO_3_) and sulfuric acid (H_2_SO_4_) are generated, which can be known as the secondary source of pollutants. The abnormal deposition of these secondary contaminants causes the acidification of water sources and damages agricultural crops^6,7^. Therefore, the separation of these detrimental pollutants has found paramount importance due to the legislation of strict environmental regulations all over the world^8,9^.

Membrane-based gas absorption using microporous hollow fiber membrane contactor (HFMC) is known as a trustworthy alternative for prevalent separation processes of greenhouse gases such as cryogenic distillation, packed bed tower, spray tower and adsorption^10–14^. HFMCs have been recently of great interest as a mass transfer device due to having disparate advantages like constant interfacial areas, flexibility of operation, and simplicity of scale-up and independent adjustment of gas/ liquid streams^15–18^. The important role of membrane materials in the separation of acidic pollutants is incontrovertible. In recent years, polypropylene (PP), polyvinylidene fluoride (PVDF), polyether sulfone (PSf) and polytetrafluoroethylene (PTFE) are among the most commonly-employed materials for fabricating hydrophobic membranes^13,19–23^. True selection of chemical absorbent is an important responsibility of researchers and scientists to improve the efficiency of greenhouse gases separation. The existence of some advantages such as eco-friendliness, suitable selectivity, excellent efficiency and reasonable cost may increase the popularity of a liquid absorbent for using in the membrane-based gas absorption processes^14,24^.

CFD is a new branch of science, which possesses great capability to predict the fluid-flow phenomena on the basis of the conservation laws^25–27^. Due to the indisputable benefit of the CFD approach for different process industries, its rapid advancement and vast utilizations have taken place during the recent decades^28,29^. Continuous development of CFD tools and its growing ability to predict the results with lower costs have significantly increased the popularity of this approach among the researchers of different scientific scopes^30–34^. In the case of membrane-based gas absorption process, the application of CFD technique to analyze the principal transport equations through disparate sides of HFMC has been a promising alternative to decrease the cost of experimental investigations^35–37^.

In an interesting study, Ariono et al. employed PP membrane contactor for the separation of SO_2_ from flue gas. Based on their simulation results, Na_2_SO_3_ aqueous solution was introduced as a promising liquid absorbent and could 1.8 times improve the absorption flux of SO_2_ acidic gas compared to water^38^. In another investigation, Kong et al. numerically analyzed the mass transfer performance of a ceramic HFMC for the separation of post-combustion SO_2_ from flue gas using NaOH alkali absorbent. They concluded that at a constant gas velocity (500 mm.s^−1^) and SO_2_ concentration (0.041 mol.m^−3^), increment in the concentration of NaOH from 0.2 to 1.5 mol. L^−1^ significantly enhanced the SO_2_ flux from 0.1 to about 0.55 mol.m^−2^ h^−1^^39^.

This article aims to develop a numerical simulation based on the CFD and finite element (FE) techniques to predict the separation percentage of SO_2_ greenhouse gas from a gaseous flow containing SO_2_ and air inside the HFMC. As the novelty, three liquid absorbents (dimethyl aniline (DMA), sodium hydroxide (NaOH) and seawater (H_2_O)) are compared with each other and the most effective one is introduced. Ultimately, investigating the effect of important operational and also membrane/module parameters on the SO_2_ separation is investigated as another highlight of this research paper.

## Reaction mechanism of SO_2_ in different absorbents

**Figure 1:**
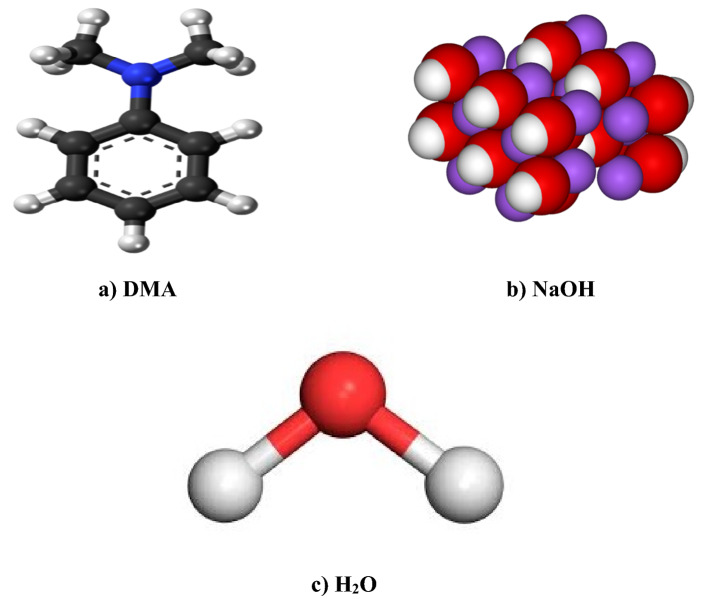
Ball-and-stick molecular structure of employed liquid absorbents^40–42^.

The ball-and-stick molecular structure of employed liquid absorbents (DMA, NaOH and H_2_O) is presented in Fig. 1.

The chemical absorption of SO_2_ acidic contaminant in H_2_O absorbent takes place by a hydrolysis reaction, presented by the equilibriums 1 to 4^7^.

1 $$SO_{2} \left( g \right) \leftrightarrow SO_{2} \left( {aq} \right)$$

2 $$SO_{2} + H_{2} O \leftrightarrow H_{2} SO_{3}$$

3 $$H_{2} SO_{3} \leftrightarrow H^{ + } + HSO_{3}^{ - }$$

4 $$HSO_{3}^{ - } \leftrightarrow H^{ + } + SO_{3}^{2 - }$$

Seawater can be considered as a complex system, which includes disparate dissolved chemical components like Na^+^, Mg^2+^, Ca^2+^, K^+^, SO_4_^2−^ and HCO_3_^−^ and Br^−^. These components contain more than 95% of dissolved salt in seawater. Additionally, NaCl has a significant value in seawater and occupies nearly 85% of the constant component^8^. The existed carbonate system in the seawater can be obviously described by the following equilibriums^18,43,44^:

5 $$H_{2} O + CO_{2} \leftrightarrow H_{2} CO_{3}$$

6 $$H_{2} CO_{3} \leftrightarrow H^{ + } + HCO_{3}^{ - }$$

7 $$HCO_{3}^{ - } \leftrightarrow H^{ + } + CO_{3}^{2 - }$$

8 $$CaCO_{3} \leftrightarrow Ca^{2 + } + CO_{3}^{2 - }$$

9 $$MgCO_{3} + Mg^{2 + } + CO_{3}^{2 - }$$

10 $$NaCO_{3}^{ - } \leftrightarrow Na^{ + } + CO_{3}^{2 - }$$

11 $$KCO_{3}^{ - } \leftrightarrow K^{ + } + CO_{3}^{2 - }$$

12 $$CaHCO_{3}^{ + } \leftrightarrow Ca^{2 + } + HCO_{3}^{ - }$$

13 $$MgHCO_{3}^{ + } \leftrightarrow Mg^{2 + } + HCO_{3}^{ - }$$

14 $$NaHCO_{3} \leftrightarrow Na^{2 + } + HCO_{3}^{ - }$$

15 $$KHCO_{3} \leftrightarrow K^{ + } + HCO_{3}^{ - }$$

According to the abovementioned equilibriums, seawater possesses great potential for the absorption of SO_2_ acidic pollutant. The presence of complex CO_2_–H_2_O–HCO_3_^−^–CO_3_^2−^ equilibrium system eventuates in increasing the mass transfer performance of SO_2_ acidic pollutant in the seawater, which positively encourages its removal.

In the case of SO_2_ separation using DMA liquid absorbent, the following reactions take place^45^:

16 $$SO_{2} + H_{2} O \leftrightarrow H^{ + } + HSO_{3}^{ - }$$

17 $$HSO_{3}^{ - } \leftrightarrow H^{ + } + SO_{3}^{2 - }$$

18 $$DMA + H^{ + } \leftrightarrow DMAH^{ + }$$

Moreover, formation of an additional compound occurs during the SO_2_ − DMA reaction as follows^46^:

19 $$SO_{2} + C_{6} H_{5} N\left( {CH_{3} } \right)_{2} \leftrightarrow C_{6} H_{5} N\left( {CH_{3} } \right)_{2} .SO_{2}$$

The separation process of SO_2_ acidic pollutant in the NaOH occurs by the following equilibrium^47^:

20 $$NaOH + SO_{2} \leftrightarrow Na_{2} SO_{3} + H_{2} O$$

## Modeling

**Figure 2:**
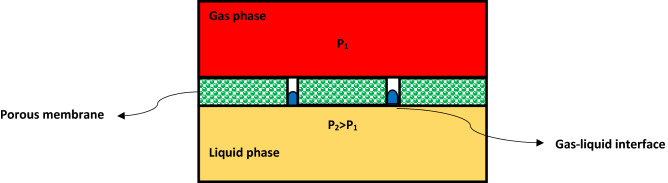
Schematic depiction of gas–liquid interface inside a microporous HFMC.

HFMC is a novel apparatus, which is designed to carry out the separation process using a hydrophobic microporous membrane^48^. Employed membrane in a gas–liquid HFMC is usually applied as a gas–liquid interface and provide better chance for efficient contact between two phases without direct mixing^49,50^. Great selectivity of the HFMC is due to the existence of gradient between the components’ solubility in the liquid phase. Thus, the majority of commercial HFMC apply microporous membranes due to having higher mass transfer properties^51^. Figure 2 schematically shows the gas–liquid interface inside a microporous HFMC.

**Figure 3:**
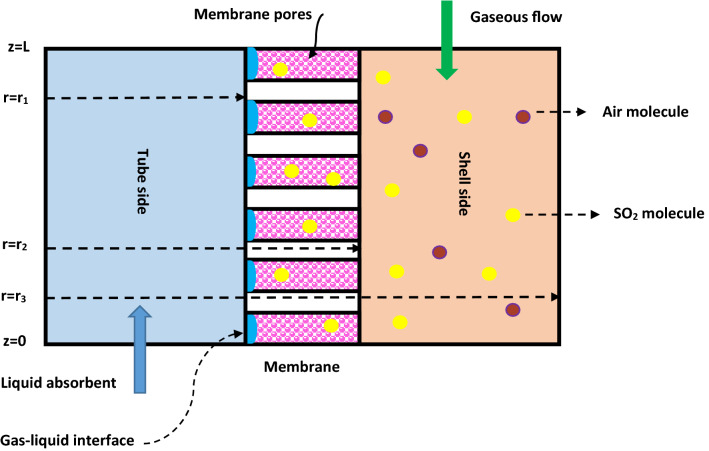
2D depiction of SO_2_ mass transfer inside different domains of contactor.

Through each HFMC, the gas–liquid mass transfer process takes place via the mechanism of solution diffusion inside the micropores of a hydrophobic membrane. Figure 3 schematically presents the two-dimensional (2D) illustration of SO_2_ mass transfer inside different domains (shell, membrane and tube) of HFMC.

As can be seen in Fig. 3, gaseous mixture including SO_2_ air flows in the shell side form up to down (from z = L to z = 0) and H_2_O, DMA and NaOH liquid absorbents move in the tube side from down to top (from z = 0 to z = L), counter-currently. The employed assumptions to implement the mathematical modeling and 2D simulation is presented in Table 1.

**Table 1:** Applied assumptions to develop the simulation^52–57^.

No	Assumption
1	Steady state operation
2	Non-wetting mode in membrane (micropores is only filled with gas molecules)
3	Isothermal condition of gas and liquid streams
4	The use of Happel’s free surface model for approximating the assumptive radius of shell around each fiber
5	Ideal behavior of gas and liquid absorbent
6	Hydrophobicity nature of employed membrane
7	Laminar flow regime in both tube and shell
8	The use of Henry's law in the membrane-liquid contact interface

COMSOL Multiphysics is as an attractive CFD-based software with brilliant capability to solve partial differential equations with different stiff/non-stiff boundary and initial conditions. In this paper, PDEs of mass and momentum are solved using this robust software based on CFD approach. To solve PDEs of mass and momentum, COMSOL Multiphysics version 6 was installed on a 64-bit platform with an Intel(R) core (TM) i7-10510U CPU and a 16 Gigabyte RAM. The needed time for solving the PDEs and present the results was about 20 s. Moreover, with the aim of managing the material balance error during the solution of mass/momentum PDEs, PARDISO numerical solver was employed owing to its brilliant advantages like excellent memory performance and robustness^58,59^. The principal PDEs of mass and momentum in tube, membrane and shell sides are presented in Table 2.

**Table 2:** Applied governing PDEs in different compartments of HFMC for developing the model^60–62^.

Mass transfer equation		
*Domain*	Tube	$D_{i,t} \left[ {\frac{{\partial^{2} C_{i,t} }}{{\partial r^{2} }} + \frac{1}{r}\frac{{\partial C_{i,t} }}{\partial r} + \frac{{\partial^{2} C_{i,t} }}{{\partial z^{2} }}} \right] + R_{i} = V_{z,t} \frac{{\partial C_{i,t} }}{\partial z}\;\;\;(21)$
	Shell	$D_{{SO_{2} , s}} \left[ {\frac{{\partial^{2} C_{{SO_{2} ,s}} }}{{\partial r^{2} }} + \frac{1}{r}\frac{{\partial C_{{SO_{2} ,s}} }}{\partial r} + \frac{{\partial^{2} C_{{SO_{2} ,s}} }}{{\partial z^{2} }}} \right] = V_{z,s} \frac{{\partial C_{{SO_{2} ,s}} }}{\partial z}\;\;\;(22)$
	Membrane	$D_{{SO_{2} ,mem}} \left[ {\frac{{\partial^{2} C_{{SO_{2} ,mem}} }}{{\partial r^{2} }} + \frac{1}{r}\frac{{\partial C_{{SO_{2} ,mem}} }}{\partial r} + \frac{{\partial^{2} C_{{SO_{2} ,mem}} }}{{\partial z^{2} }}} \right] = 0\;\;\;(23)$
Momentum equation		
*Domain*	Shell	$V_{z,s} = 2\overline{{V_{s} }} \left[ {1 - \left( {\frac{{r_{2} }}{{r_{3} }}} \right)^{2} } \right] \times \left[ {\frac{{(r/r_{3} )^{2} - (r_{2} /r_{3} )^{2} + 2\ln \left( {r_{2} /r} \right)}}{{3 + (r_{2} /r_{3} )^{4} - 4(r_{2} /r_{3} )^{2} + 4\ln \left( {r_{2} /r_{3} } \right)}}} \right]\;\;\;(24)$
	Tube	$V_{z,t} = 2\overline{{V_{t} }} \left[ {1 - \left( {\frac{r}{{r_{1} }}} \right)^{2} } \right]\;\;\;(25)$

In Table 2, ${D}_{{SO}_{2}, s}$,${D}_{{SO}_{2},mem}$ are defined as the diffusion coefficient of SO_2_ greenhouse gas in the shell and membrane. Also, ${D}_{i,t}$ is the diffusion coefficient of i (SO_2_, H_2_O, NaOH and DMA) in the tube. Additionally, *V*_*z,s*,_ ${V}_{z,t},\overline{{ V }_{s}}$,$\overline{{V }_{t}}$ and *C* are described as the shell’s velocity in axial direction, tube velocity in the axial direction, the average velocity in the shell, the average velocity inside the tube and concentration, respectively. Boundary conditions at each main domains of HFMC are presented in Table 3.

**Table 3:** Employed boundary/initial conditions in different compartments of HFMC.

Position	Shell	Membrane	Tube
r = 0	–	–	$\partial {\text{C}}_{{{\text{SO}}_{2} ,{\text{t}}}} /\partial {\text{r}} = 0$
r = r_1_	–	C_mem_ = C_t_/m	C_t_ = C_mem_*m
r = r_2_	C_s_ = C_mem_	C_mem_ = C_s_	–
r = r_3_	${ }\partial {\text{C}}_{{{\text{SO}}_{2} ,{\text{s}}}} /\partial {\text{r}} = 0$	–	–
z = 0	${\text{C}}_{{{\text{SO}}_{2} ,{\text{s}}}} = {\text{C}}_{{\text{initial }}}$	Insulated	Outlet flow
z = L	Outlet flow	Insulated	${\text{C}}_{{{\text{SO}}_{2} ,{\text{t}}}} = 0$ ${\text{C}}_{{{\text{solution}},{\text{t}}}} = {\text{C}}_{{\text{initial }}}$

The required parameters of microporous membrane and module following with important physicochemical properties of SO_2_ acidic pollutants and seawater, NaOH and DMA liquid absorbents are rendered in Table 4.

**Table 4:** Membrane/module parameters and physicochemical properties of SO_2_, seawater, NaOH and DMA liquid absorbents.

Parameter	Unit	Value	Ref
Fibers I.D	m	2.410^−4^	^63^
Fibers O.D	m	310^−4^	^63^
Shell O.D	m	0.457210^−2^	^63^
Porosity of membrane ($\upvarepsilon$)	–	0.4	^63^
Tortuosity of membrane ($\uptau$)		2.5	^63^
Fiber’s length (L)	m	0.18	^63^
Number of fibers (n)	–	75	^63^
${\mathrm{D}}_{{\mathrm{SO}}_{2},\mathrm{g}}$	m^2^.s^−1^	1.2610^−5^	^64^
${\mathrm{D}}_{{\mathrm{SO}}_{2},\mathrm{mem}}$	m^2^.s^−1^	${D}_{S{O}_{2},g}(\frac{\upvarepsilon }{\uptau })$	^65^
${\mathrm{D}}_{{\mathrm{SO}}_{2},{\mathrm{H}}_{2}\mathrm{O}}$	m^2^.s^−1^	210^−9^	^66^
${\mathrm{D}}_{{\mathrm{SO}}_{2},\mathrm{DMA}}$	m^2^.s^−1^	2.110^−9^	^67^
${\mathrm{D}}_{{\mathrm{SO}}_{2},\mathrm{NaOH}}$	m^2^.s^−1^	2.12210^−9^	^68^
${\mathrm{m}}_{{\mathrm{SO}}_{2},{\mathrm{H}}_{2}\mathrm{O}}$	–	25.86	^69^
${\mathrm{m}}_{{\mathrm{SO}}_{2},\mathrm{DMA}}$	–	0.00131	^70^
${\mathrm{m}}_{{\mathrm{SO}}_{2},\mathrm{NaOH}}$	–	0.952	^71^

*ID* Inner diameter; *OD* Outer diameter.

## Results and discussion

### Validation of developed modeling and 2D simulation

**Figure 4:**
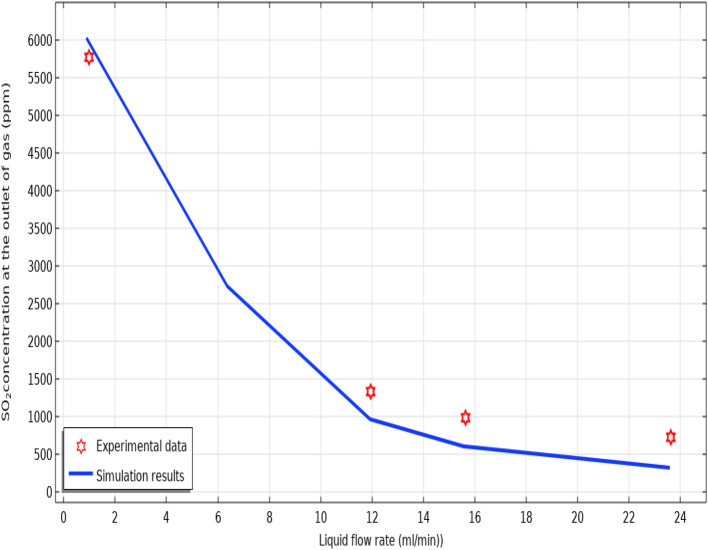
Validation of developed 2D simulation with experimental results. Feed gas composition: 1% SO_2_ in air T = 298 K, Q_g_ = 200 ml min^−1^. Experimental data was according to the study of Karoor and Sirkar^63^.

Up to our knowledge, very few papers experimentally evaluated the performance of NaOH, DMA and H_2_O to separate SO_2_ acidic pollutant. Therefore, the validation of developed 2D simulation was performed via the comparison of simulation outcomes with experimental results obtained by Karoor and Sirkar about the separation of SO_2_ using pure water^63^. As demonstrated in Fig. 4, there is a favorable agreement between the experimental data and predicted results achieved by 2D simulation, which corroborates the accuracy and validation of employed modeling and simulation in this work.

**Figure 5:**
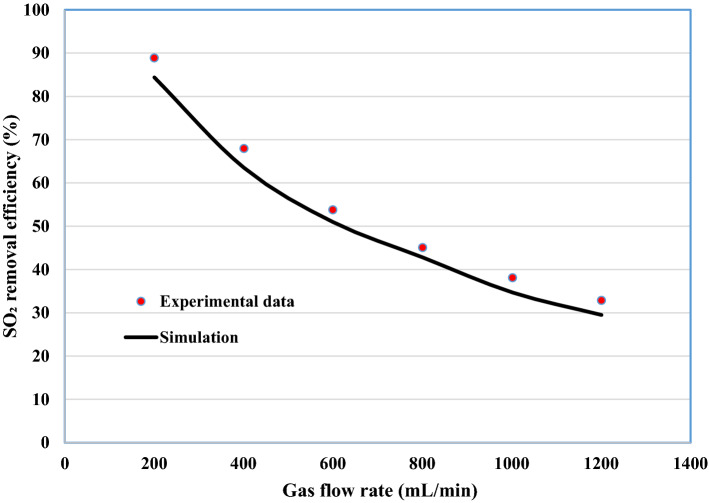
Validation of developed 2D simulation with experimental results. Inlet SO_2_ concentration: 1000 ppm, T = 298 K, Q_l_ = 25 ml min^−1^. Experimental data was according to the study Xu et al.^72^.

With the aim of ensuring the accuracy of developed model outcomes, the second validation was implemented via the comparison of simulation results with obtained experimental data from Xu et al. for the separation of SO_2_ using NaOH solution^72^. As can be seen in Fig. 5, an excellent agreement is again demonstrated between the experimental data and predicted results with the absolute relative deviation (ARD) of about 4%, which certainly corroborates the validation of developed model.

### Axial concentration gradient of SO_2_

**Figure 6:**
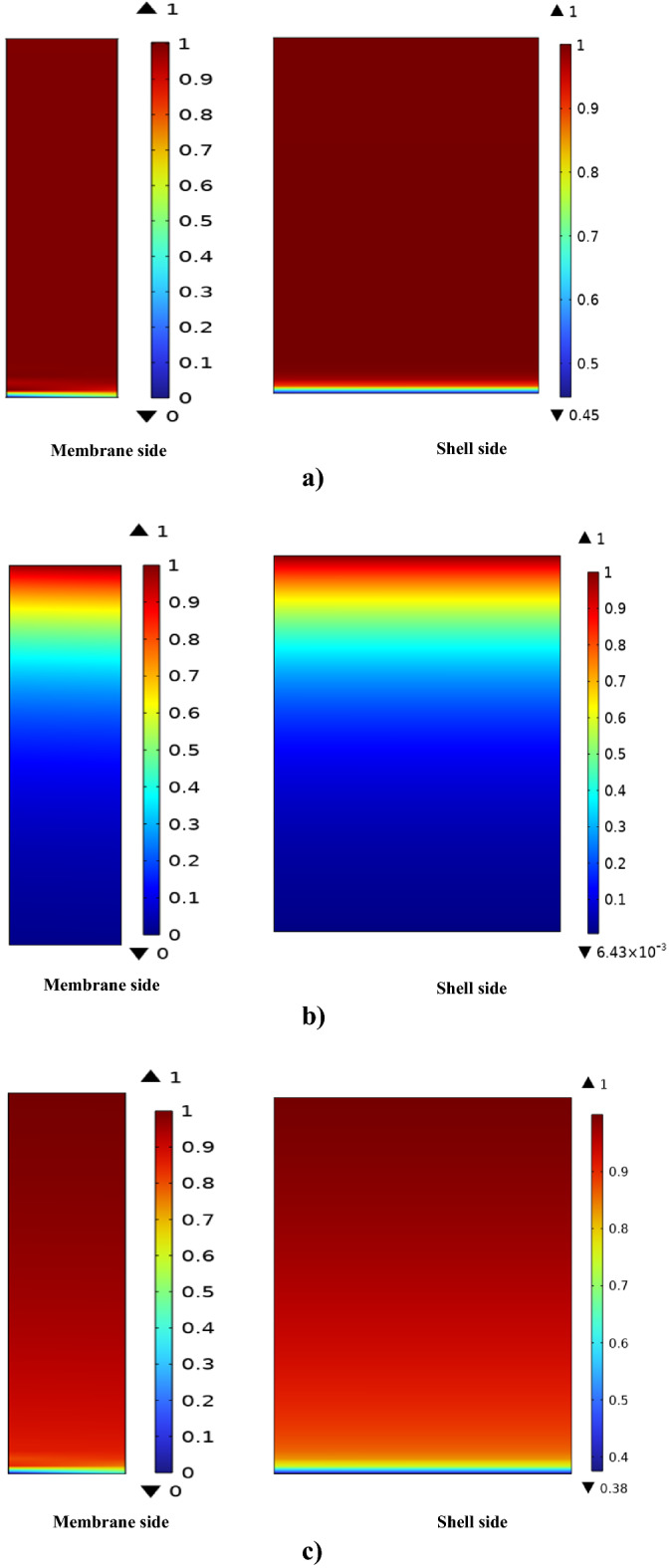
Axial concentration gradient of SO_2_ greenhouse gas in the shell and membrane sides of the contactor using (**a**) DMA, (**b**) H_2_O and (**c**) NaOH liquid absorbents.

Figures 6a, 6b and 6c show the axial concentration profile of SO_2_ greenhouse gas in the shell and membrane sides of the contactor, respectively. The SO_2_/air gaseous mixture enters to the shell side of HFMC from z = L, where the SO_2_ concentration is maximum. On the other hand, employed liquid absorbents (NaOH, H_2_O and DMA) enters to the tube compartment of contactor from z = 0. At this location, the concentration of liquid absorbents is in the highest value and the concentration of SO_2_ is considered zero. When the gaseous mixture flows inside the shell, the presence of concentration difference causes the movement of SO_2_ from shell to the porous membrane. Diffusion of SO_2_ molecules to the membrane micropores facilitates their contact and consequently their removal by flowing absorbents in the tube side. The results show that the dimensionless concentration of SO_2_ in the shell side declines from 1 to 0.45, 0.0064 and 0.38 using DMA, seawater and NaOH, respectively. This finding proves the SO_2_ separation percentage of 55, 99.36 and 62% by employing DMA, seawater and NaOH as liquid absorbents. High SO_2_ separation efficiency of seawater can be owing to the presence of complex CO_2_–H_2_O–HCO_3_^−^–CO_3_^2−^ equilibrium system, which significantly enhances its mass transfer coefficient compared to other liquid absorbents^8^.

### The role of gas and liquid flow rates on the SO_2_ separation performance

**Figure 7:**
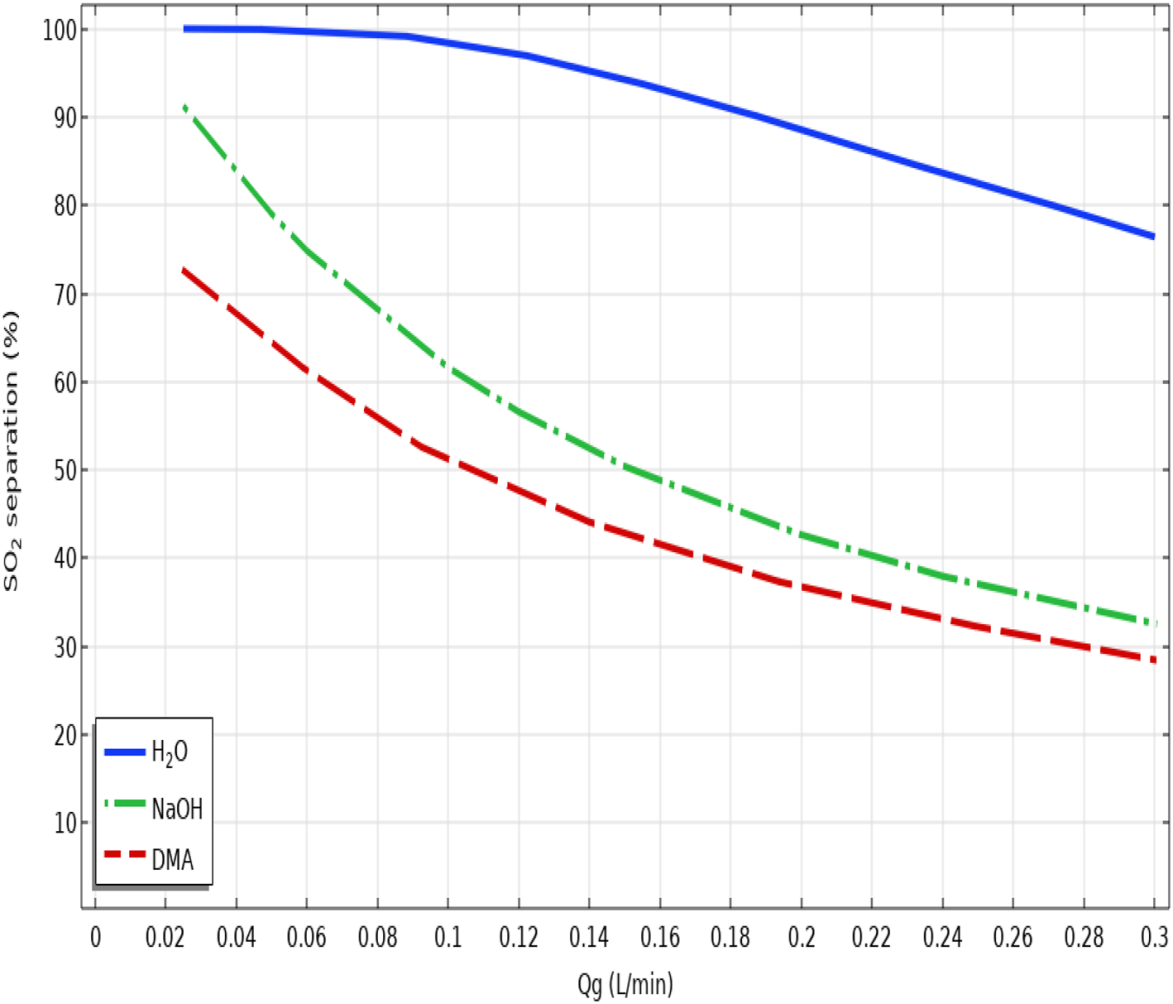
Effect of gas flow rate on the separation yield of SO_2_ greenhouse gas.

Figure 7 illustrate the impact of gas flow rate on the separation yield of SO_2_. As presented, increase in the flow rate of gaseous mixture considerably declines the residence time in the module. As the result, decrement of residence time destroys the suitable contact of SO_2_ with liquid absorbents, which results in decreasing the separation efficacy. By looking at the figure, it is perceived that increase in the gas flow rate from 0.25 to 0.3 L/min decreases the SO_2_ separation percentage from 100 to 77% using seawater, from 91 to 33% using NaOH and from 72 to 29% using DMA. Table 5 enlists the separation percentage of SO_2_ from the gaseous mixture using seawater, NaOH and DMA liquid absorbents in different gas flow rates.

**Table 5:** SO_2_ separation percentage using seawater, NaOH and DMA liquid absorbents in disparate gas flow rates.

Gas flow rate (L.min^−1^)	SO_2_ separation using seawater (%)	SO_2_ separation using NaOH (%)	SO_2_ separation using DMA (%)
0.025	100	91.22	72.67
0.1	98.34	61.57	51.14
0.16	93.04	48.65	42.69
0.2	88.57	42.52	36.72
0.25	82.6	37.06	32.09
0.3	76.31	32.42	28.28

**Figure 8:**
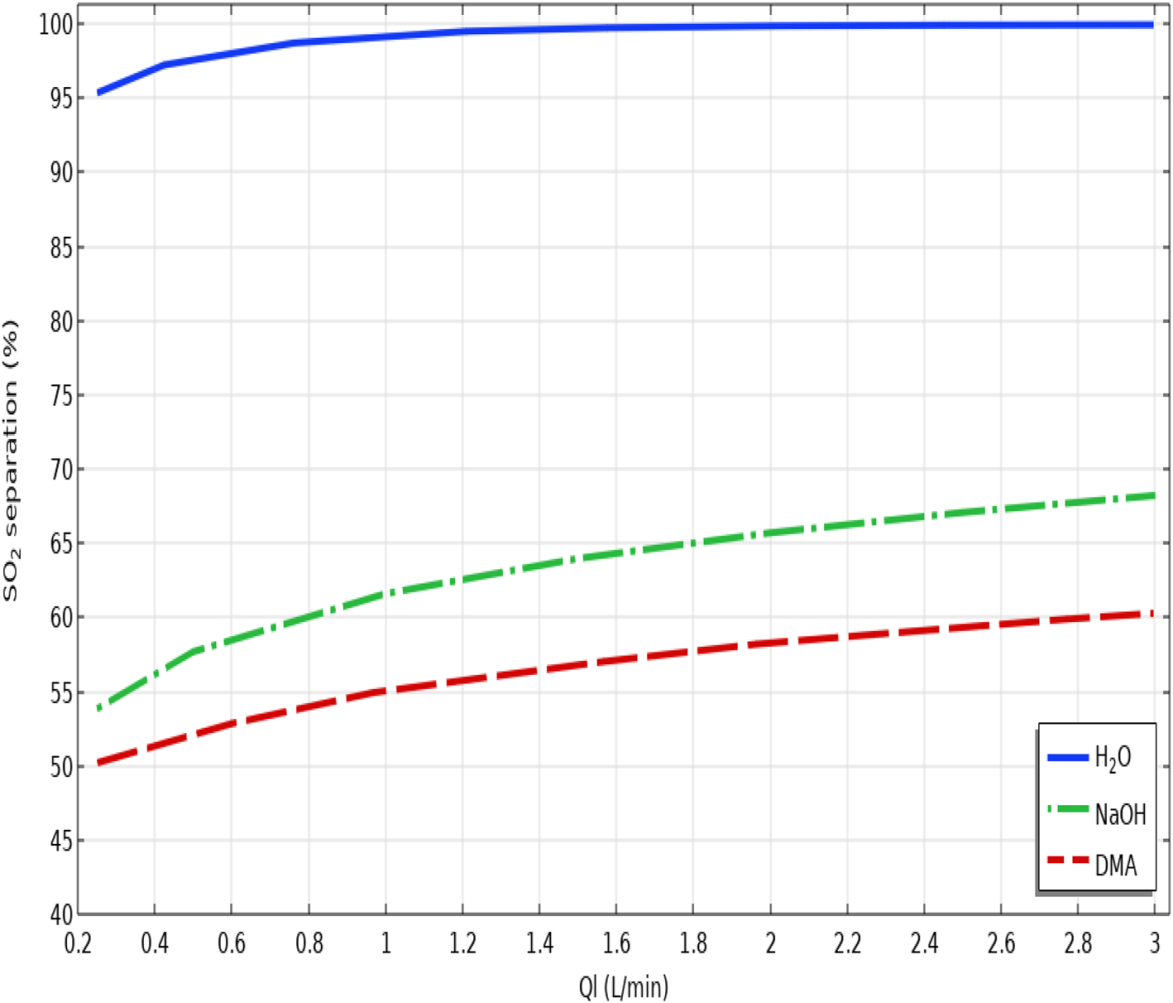
Effect of liquid absorbents’ flow rate on the separation yield of SO_2_ greenhouse gas.

Additionally, the influence of liquid absorbents’ flow rate on the percentage of SO_2_ separation is shown in Fig. 8. By faster flowing of absorbents through the tube segment, the concentration of gas at the external surface of the hollow fiber along the length of the HFMC decreases significantly, which eventuates in greater mass transfer coefficient, superior concentration gradient at the shell-membrane interface and therefore, better SO_2_ separation performance. Based on the figure, increase in the flow rate of liquid absorbents from 0.25 to 0.3 L/min improves the SO_2_ separation percentage from 95 to 100% using seawater, from 53.5 to about 67.5% using NaOH and from 50 to 60% using DMA.

The separation performance of SO_2_ greenhouse pollutant from SO_2_/air gaseous stream applying seawater, NaOH and DMA absorbents in different gas flow rates is presented in Table 6.

**Table 6:** SO_2_ separation performance from SO_2_/air gaseous stream using seawater, NaOH and DMA liquid absorbents in different liquid flow rates.

Liquid flow rate (L.min^−1^)	SO_2_ separation using seawater (%)	SO_2_ separation using NaOH (%)	SO_2_ separation using DMA (%)
0.25	95.38	53.92	50.19
1	99.11	61.59	55.1
1.6	99.8	64.34	56.77
2	99.85	65.71	58.35
2.5	99.9	67.09	59.33
3	99.95	68.27	60.31

### Effect of membrane/module specifications on the separation performance

**Figure 9:**
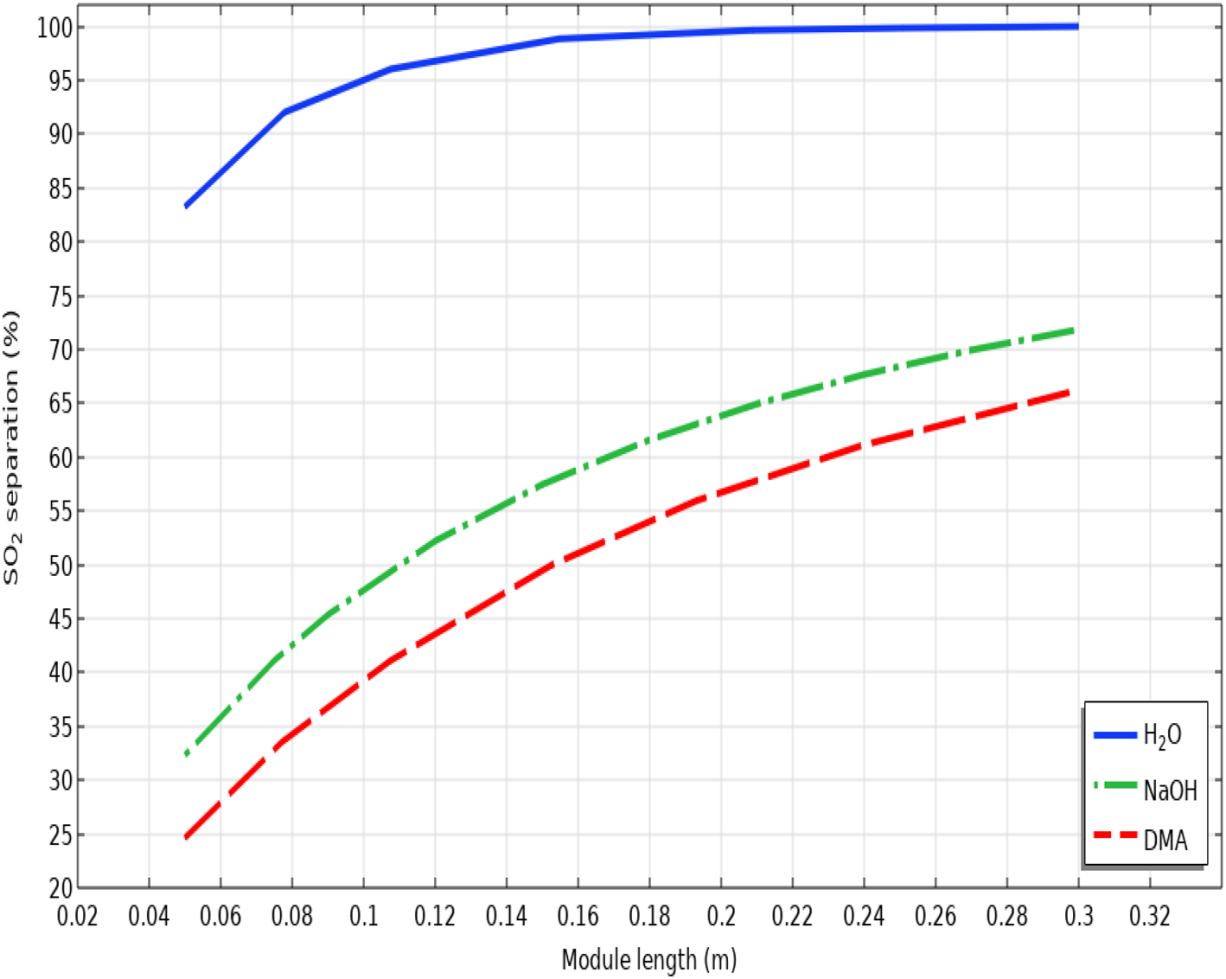
Effect of module length on the separation yield of SO_2_ greenhouse gas.

Figure 9 presents a schematic demonstration for evaluating the role of module length on the separation yield of SO_2_ greenhouse gas. As illustrated, increase in the length of module possesses positive impact on improving the gas-absorbent residence time and contact area between two phases, which results in enhancing the separation yield of SO_2_ greenhouse gas. It is observed that increase in the length of module from 0.05 to 0.3 m improves the SO_2_ separation percentage from 83 to 100% using seawater, from 32.5 to about 72% using NaOH and from 25 to 66% using DMA.

The separation percentage of SO_2_ from gaseous flow in different length of module is presented in Table 7.

**Table 7:** SO_2_ separation performance using seawater, NaOH and DMA liquid absorbents in different lengths of module.

Module length (m)	SO_2_ separation using seawater (%)	SO_2_ separation using NaOH (%)	SO_2_ separation using DMA (%)
0.05	83.14	32.3	24.54
0.15	98.52	57.45	49.56
0.2	99.46	63.87	56.65
0.25	99.73	68.42	62
0.3	99.86	71.77	66.15

**Figure 10:**
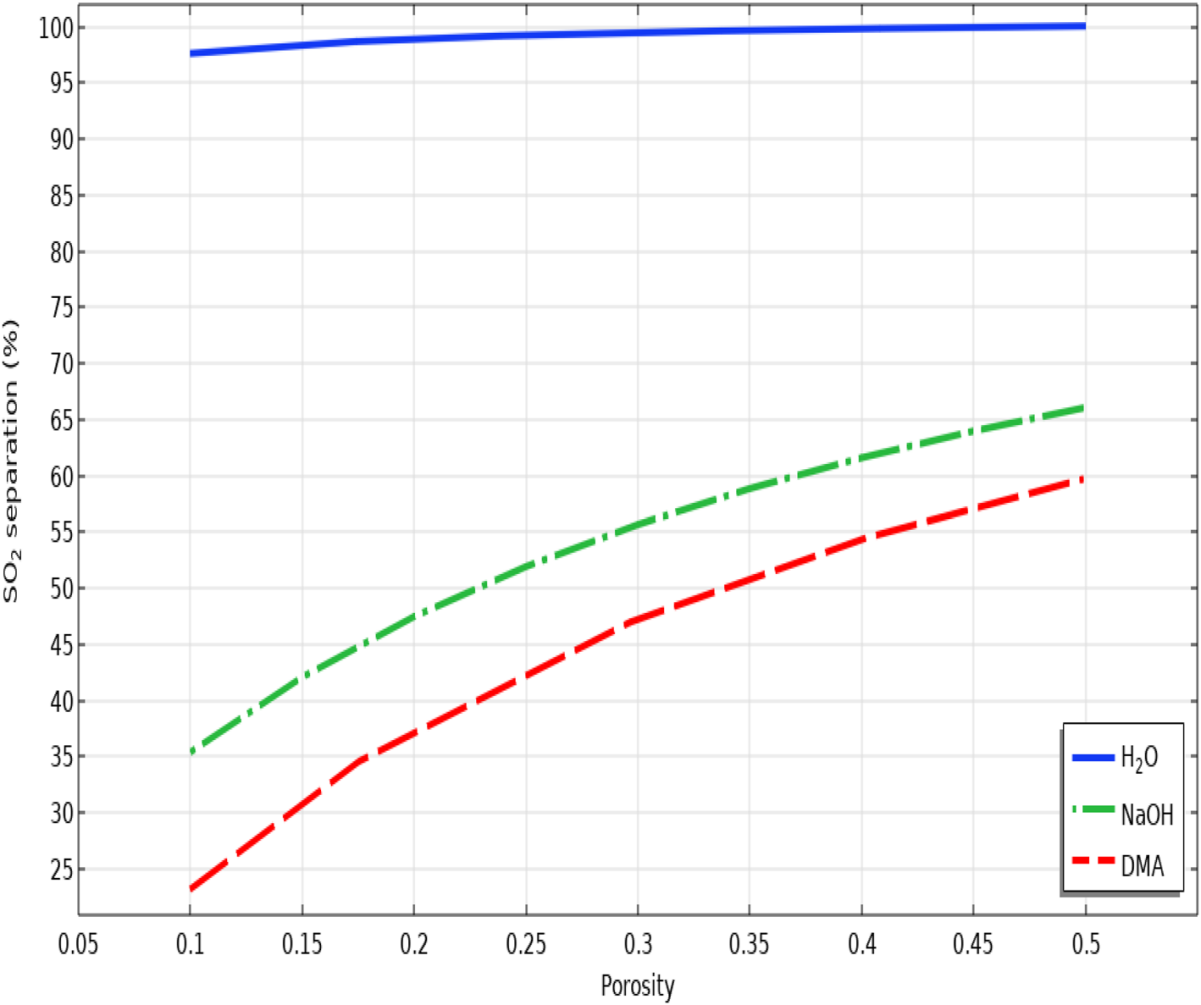
Effect of membrane porosity on the separation yield of SO_2_ greenhouse gas.

Membrane porosity is a membrane-related parameter, which its increment may have an encouraging influence on the separation performance of various greenhouse gases. As shown in Fig. 10, increase in the porosity of polypropylene membrane from 0.1 to 0.5 cause a substantial enhancement in the removal efficacy of SO_2_ greenhouse gas from 97 to 100% using seawater, from 35 to about 66% using NaOH and from 23 to 60% using DMA. This substantial increment can be justified due to this reality that increase in the porosity of membrane results in the enhancement of SO_2_ diffusivity in the fiber micropores and also the deterioration of the mass transfer resistance inside the HFMC. Table 8 aims to present a data analysis about the role of porosity on increasing the separation percentage of SO_2_ using employed chemical absorbents in the HFMC.

**Table 8:** Impact of membrane porosity on the SO_2_ separation performance using seawater, NaOH and DMA liquid absorbents.

Porosity	SO_2_ separation using seawater (%)	SO_2_ separation using NaOH (%)	SO_2_ separation using DMA (%)
0.1	96.98	18.29	3.02
0.2	98.65	33.72	20.63
0.3	99.49	43.96	33.38
0.4	100	51.51	42.28
0.5	100	57.21	49.16

**Figure 11:**
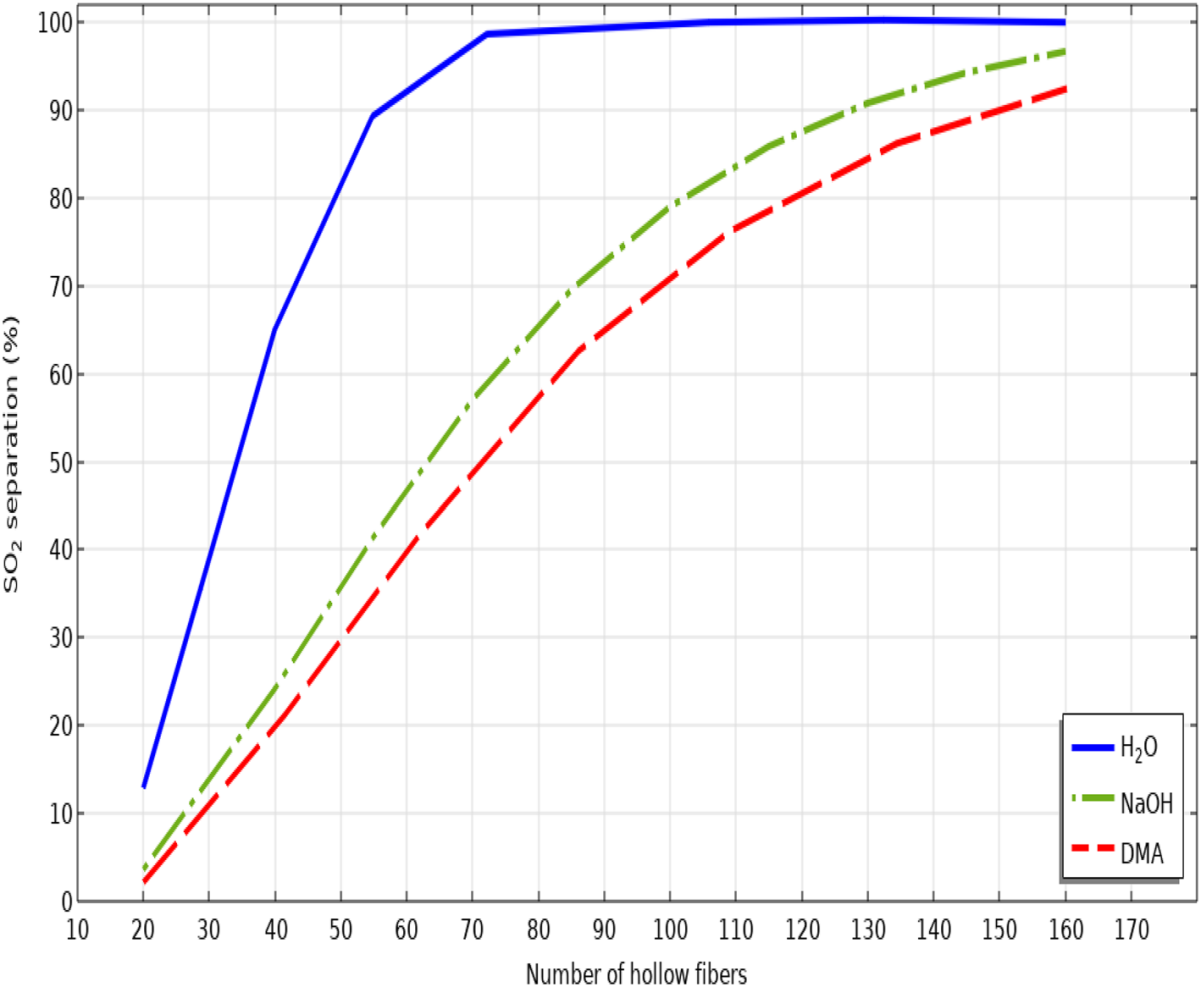
Effect of the number of hollow fibers on the separation yield of SO_2_ greenhouse gas.

Figure 11 schematically presents the effect of hollow fibers’ number on the separation of SO_2_ greenhouse pollutant. As would be expected, increase in the number of microporous fibers substantially improves the gas-absorbent mass transfer interface and also their related contact area. Increase in the gas–liquid mass transfer interface and their contact area significantly increases the mass transfer coefficient of SO_2_ and therefore, its separation percentage. It is demonstrated that increase in the number of fibers from 20 to 160 improves the SO_2_ separation percentage from 13 to 100% using seawater, from 4 to about 96% using NaOH and from 2 to 92% using DMA.

Table 9 comprehensively presents the separation efficiency of SO_2_ greenhouse contaminant in different number of hollow fibers.

**Table 9:** Impact of the number of fibers on the SO_2_ separation performance using seawater, NaOH and DMA liquid absorbents.

Number of fibers	SO_2_ separation using seawater (%)	SO_2_ separation using NaOH (%)	SO_2_ separation using DMA (%)
20	13.15	3.89	2.72
40	65.1	24.51	19.96
60	92.045	46.75	40.1
80	99.025	65.42	57.3
100	99.67	79.05	70.77
120	100	87.5	80.52
140	100	93.01	87.5
160	100	96.6	92.37

## Conclusion

Over the last decades, industrial application of HFMCs to alleviate the extraordinary release of various greenhouse gases like SO_2_ to the atmosphere has been of great attention. In this paper, the removal performance of SO_2_ greenhouse contaminant form SO_2_/air mixture using three novel liquid absorbents (seawater (H_2_O), DMA and NaOH) was evaluated inside the HFMC. To reach this aim, a CFD-based comprehensive simulation was developed to predict the results. An FE-based mathematical model was also applied to solve the PDEs of transport in the main subdomains of contactor. The results corroborated that seawater can be recommended as the most efficacious liquid absorbent for removing SO_2_ with the removal efficiency of around 99.36%. After seawater, NaOH and DMA were placed at the second and third rank with the SO_2_ separation percentage of 62 and 55%, respectively (seawater (H_2_O) > NaOH > DMA). Evaluation of simulation outcomes proved the deteriorative impact of gas flow rate on the SO_2_ separation yield (due to decreasing the residence time of gaseous mixture in the HFMC). But, increment of other parameters like absorbent’s flow rate, length of membrane module, hollow fibers’ number and porosity possesses encouraging influence on the separation performance of SO_2_ acidic pollutant due to declining the concentration of gaseous mixture at the external surface of hollow fibers, increasing the gas-absorbent contact area, increasing the diffusivity of SO_2_ and improving the gas–liquid residence time inside the contactor, respectively.

## Data Availability

All data generated or analyzed during this study are included in this published article.
